# Significance of GABA_A_ Receptor for Cognitive Function and Hippocampal Pathology

**DOI:** 10.3390/ijms222212456

**Published:** 2021-11-18

**Authors:** Yuya Sakimoto, Paw Min-Thein Oo, Makoto Goshima, Itsuki Kanehisa, Yutaro Tsukada, Dai Mitsushima

**Affiliations:** Department of Physiology, Yamaguchi University Graduate School of Medicine, Ube 755-8505, Japan; pawmto@gmail.com (P.M.-T.O.); b905eb@yamaguchi-u.ac.jp (M.G.); i029eb@yamaguchi-u.ac.jp (I.K.); i057@yamaguchi-u.ac.jp (Y.T.); mitsu@yamaguchi-u.ac.jp (D.M.)

**Keywords:** AMPA receptor, GABA_A_ receptor, contextual learning, synaptic plasticity

## Abstract

The hippocampus is a primary area for contextual memory, known to process spatiotemporal information within a specific episode. Long-term strengthening of glutamatergic transmission is a mechanism of contextual learning in the dorsal cornu ammonis 1 (CA1) area of the hippocampus. CA1-specific immobilization or blockade of α-amino-3-hydroxyl-5-methyl-4-isoxazole-propionate (AMPA) receptor delivery can impair learning performance, indicating a causal relationship between learning and receptor delivery into the synapse. Moreover, contextual learning also strengthens GABA_A_ (gamma-aminobutyric acid) receptor-mediated inhibitory synapses onto CA1 neurons. Recently we revealed that strengthening of GABA_A_ receptor-mediated inhibitory synapses preceded excitatory synaptic plasticity after contextual learning, resulting in a reduced synaptic excitatory/inhibitory (E/I) input balance that returned to pretraining levels within 10 min. The faster plasticity at inhibitory synapses may allow encoding a contextual memory and prevent cognitive dysfunction in various hippocampal pathologies. In this review, we focus on the dynamic changes of GABA_A_ receptor mediated-synaptic currents after contextual learning and the intracellular mechanism underlying rapid inhibitory synaptic plasticity. In addition, we discuss that several pathologies, such as Alzheimer’s disease, autism spectrum disorders and epilepsy are characterized by alterations in GABA_A_ receptor trafficking, synaptic E/I imbalance and neuronal excitability.

## 1. Introduction

The hippocampal CA1 region has a total number of 350,000 neurons within a range from 320,000 to 380,000 at postnatal day 30 in Wistar rats [1]. Gamma-aminobutyric acid (GABA) ergic interneurons contain a conservative estimate of ~38,500 inhibitory interneurons in the CA1 region [2,3]. According to their molecular signatures, GABAergic interneurons can be divided into five main groups: Parvalbumin, somatostatin, neuropeptide Y, vasoactive intestinal peptide and cholecystokinin interneuron [4,5]. A single cornu ammonia 1 (CA1) pyramidal neuron receives approximately 3000 excitatory [6] and 1700 GABAergic synapses on their dendrites, somata and proximal axons [6]. While excitatory inputs target a distal dendric spine of a CA1 pyramidal neuron, inhibitory inputs are largely concentrated in the perisomatic region. From this distribution of excitatory and inhibitory inputs, a potent perisomatic inhibition is considered to control dendritic excitatory inputs and play an important role in the decision-making of pyramidal cell activation itself [6].

## 2. The GABAergic System

GABA is the main inhibitory neurotransmitter in the mature mammalian central nervous system. GABA is stocked in synaptic vesicles and released in the synaptic cleft after stimulation by presynaptic neuron depolarization. GABA diffuses across the cleft to target receptors on the postsynaptic region. There are three types of GABA receptors in the central nervous system, namely, ionotropic GABA_A_ and GABA_C_ receptors and metabotropic GABA_B_ receptors [5,7,8].

The nature of contextual fear learning-induced pre- and post-synaptic plasticity is complicated by the fact that learning also affects GABA_A_ receptor-mediated inhibitory synapses in CA1 pyramidal neurons [9,10,11]. GABA_A_ receptors typically consist of 2 α and 2 β subunits, together with either an γ or δ subunit [12]. Pore opening allows Cl^−^ influx to induce postsynaptic hyperpolarization upon GABA binding. Considering that each presynaptic vesicle contains ~2500 molecules of GABA [13,14], we also quantified miniature postsynaptic GABA_A_ receptor currents induced by single-synaptic GABA vesicles (miniature inhibitory postsynaptic current (mIPSC)).

The activity of GABA_A_ receptor is regulated by cross-talk with other receptors, such as NMDA receptor, dopamine D5 receptor and GABA_B_ receptor [15,16]. GABA_A_ receptors are co-localized with them in certain synapses and their neurotransmitters are simultaneously activated or co-released [15]. While co-activation of these receptors occurs with GABA_A_ receptor-suppressed GABAergic inhibition, sole GABA_A_ receptor activity inhibits the response of these receptors [15].

## 3. Contextual Fear Memory Triggers Rapid Synaptic Plasticity

Pharmacological manipulation of AMPA or GABA_A_ receptors in the CA1 suggested different roles of the receptors after training [10,17,18,19,20,21,22]. Microinjections of an AMPA receptor blocker (7-nitro-2, 3-dioxo-1, 4-dihydroquinoxaline-6-carbonitrile (CNQX)) into the CA1 impairs inhibitory avoidance (IA) task training immediately (0–5 min), but these effects are lost 30–60 min after training [17,18,21], whereas GABA_A_ receptor blocker microinjection improves performance if administered immediately after training [17,20,21,22]. While these studies suggested a critical period for plasticity immediately after training, the dynamic changes in learning-induced synaptic diversity were poorly understood. Recently, Sakimoto et. al. [23] revealed a dynamic of synaptic plasticity for memory in hippocampal CA1. Contextual learning rapidly strengthened E/I synapses in various ways in individual CA1 neurons, producing a broad diversity of synaptic input across the CA1 neuronal population within 5 min after training.

While rapid plasticity of excitatory CA1 synapses is considered an initial step of memory encoding rather than retrieval [23,24], conclusive evidence for the dynamic change of synaptic current is still lacking. We found a rapid increase in mEPSC amplitude within 5 min after IA training, showing that memory encoding rather than retrieval strengthens AMPA receptor-mediated excitatory synapses. Using fluctuation analysis of CA1 pyramidal neurons, we recently confirmed that training increased postsynaptic AMPA receptor channels without changing the cation current per channel and increase in presynaptic glutamate release [25]. As to the causal relationship between learning and plasticity, we previously reported that bilateral expression of GluA1-containing AMPA receptor delivery blockers in CA1 neurons impairs IA learning [26]. Moreover, a chromophore-assisted light-inactivation technique demonstrated that optical inactivation of synaptic AMPA receptors can erase acquired memories [27]. From these results, rapid trafficking of AMPA receptors after IA training is essential for encoding contextual memories.

**Figure 1:**
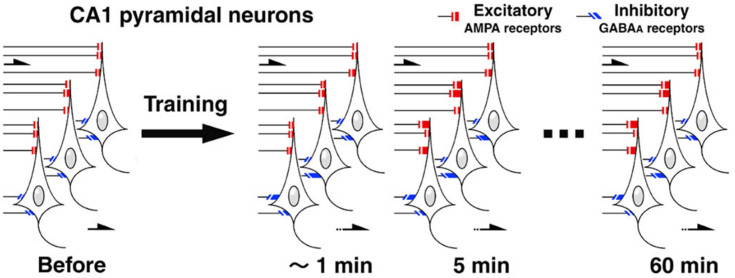
Schematic image of CA1 pyramidal neurons. IA training rapidly strengthened GABA_A_ receptor-mediated inhibitory synapses within 1 min, while the training strengthened AMPA receptor-mediated excitatory synapses within 5 min. CA1 pyramidal neurons exhibited broad diversity of excitatory/inhibitory synaptic currents within 5 min, and the neuron-specific synaptic diversity was sustained for more than 60 min.

The plasticity at inhibitory synapses seems to be task dependent and region specific [9,10,28]. As for hippocampal-dependent contextual learning, IA training clearly increased mIPSC amplitudes, suggesting postsynaptic strengthening of GABA_A_ receptor-mediated plasticity [10]. In addition, the mIPSC frequency rapidly increased without an increase in GABA release probability, suggesting a rapid activation of inhibitory silent or subthreshold synapses to increase the number of overthreshold synapses. Many mIPSC events may be small and below the detection threshold (<10 pA) and increased postsynaptic responses may increase the amplitude of these small events above the detection level (>10 pA), resulting in an apparent increase in mIPSC frequency. Moreover, Sakimoto et al. [23] found a rapid increase in mIPSC amplitude immediately after training, indicating that memory encoding rather than retrieval strengthens GABA_A_ receptor-mediated inhibitory synapses. This was the first report showing a rapid phosphorylation of the Ser^408–409^ GABA_A_ receptor β_3_ subunit (GABA_A_Rβ3) within 1 min after training, concerning sites necessary to attenuate clathrin-dependent endocytosis of synaptic receptors, leading to both increased mIPSC amplitude and frequency in cultured neurons (Figure 1) [29].

**Figure 2:**
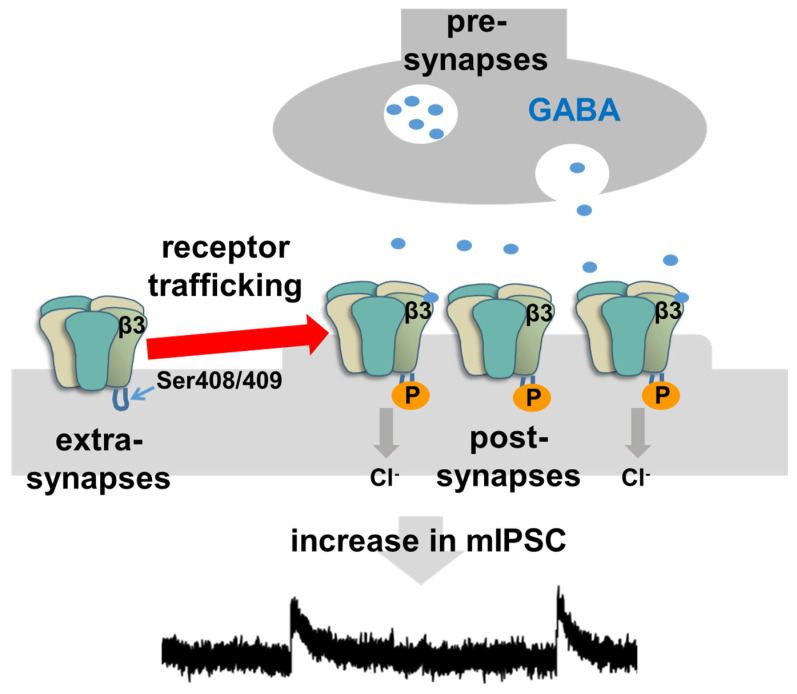
Schematic image of GABA_A_ receptor trafficking mechanisms. The phosphorylation in the β_3_ subunit Ser^408–409^ increased levels of GABA_A_ receptors at post-synapses, resulting an increase in mIPSC. Since IA training facilitated the phosphorylation in the β_3_ subunit (Ser^408–409^) and GABA_A_ receptor-mediated current within 1 min, we suggested that the rapid inhibitory plasticity may contribute to maintaining memory function in hippocampus.

A possible causal relationship between GABAergic plasticity and learning has been previously reported. Genetic deficiency of GABA_A_Rβ3 severely impairs the contextual freezing response without affecting pain perception [30], and phosphorylation in the cytoplasmic loop of the β_3_ subunit (Ser^408–409^) is known to play an essential role for PKA, PKB, PKC, Ca^2+^ and calmodulin-dependent protein kinase II-dependent plasticity [31], as phosphorylation can increase surface levels of GABA_A_ receptors containing β_3_ subunits in cultured neurons (Figure 2) [32,33,34,35]. Not only the genetic deficiency of GABA_A_Rβ3, but also prevention of GABA_A_ receptor-mediated plasticity in CA1 impairs contextual learning [10,30]. Optogenetic manipulation of CA1 neurons further proved the timing-specific causal relationship between GABAergic inputs and learning; optic inactivation of dendrite-targeting CA1 interneurons during aversive stimuli was sufficient to prevent fear teaching [11]. In a preliminary study, we found that microinjections of an interference peptide in Ser^408–409^ phosphorylation into the CA1 successfully blocked training-induced mIPSC strengthening. Moreover, bilateral peptide microinjections resulted in a drastic decrease in IA task-learning performance, suggesting further causal relationship between learning and Ser^408–409^ phosphorylation of the GABA_A_Rβ_3_ subunit.

## 4. Intracellular Mechanism of Rapid Inhibitory Synaptic Plasticity

Questions arise as to how the training can increase GABA_A_ receptor-mediated currents so rapidly. GABA_A_ receptor mobility may be closely associated with the above issue, since removal from the postsynaptic membrane or lateral diffusion decreases the synaptic GABAergic current [36,37,38]. Recent single-particle tracking analysis further demonstrated quick diffusion of a single GABA_A_ receptor (0.07 μm^2^/s) in cultured hippocampal neurons; it can move rapidly between the two synapses within a few hundred milliseconds to a few seconds. Abundant GABA_A_ receptors heterosynaptically locate at glutamatergic synapses, and play a key role in the stimulus-dependent rapid changes in the postsynaptic number of receptors [39], probably because learning may rapidly recruit heterosynaptic GABA_A_ receptors to strengthen inhibitory synapses.

Once the receptor reaches the postsynaptic region through lateral diffusion [36,37], gephyrin seems to stabilize the synaptic receptors [31,40]. Gephyrin can bind to the major subunits of GABA_A_ receptors (α_1–3_ and β_2–3_) [41] and preventing its binding decreases mIPSC amplitudes [42]. Because phosphorylation of Ser^408–409^ GABA_A_Rβ3 is known to prevent clathrin adaptor protein 2-mediated GABA_A_ receptor internalization, training-induced Ser^408–409^ phosphorylation may help to stabilize surface receptors [43,44,45]. While training-induced Ser^408–409^ phosphorylation is rapid and transient, gephyrin may contribute to sustaining large mIPSC amplitude. Finally, using fluctuation analysis of CA1 pyramidal neurons, we recently confirmed that training increases the postsynaptic number of GABA_A_ receptor channels without changing the Cl^−^ current per channel [24].

## 5. Alterations to GABA_A_Rβ3 in Cognitive Disease

Several pathologies, such as Alzheimer’s disease (AD), autism spectrum disorders (ASDs), status epilepticus (SE) and posttraumatic stress disorder (PTSD), are characterized by synaptic E/I imbalance, neuronal hyperactivity and cognitive dysfunction [30,46,47,48,49]. In particular, alterations of GABA_A_Rβ3 have been observed in all these pathologies [50].

### 5.1. AD

AD is a progressive neurologic disorder characterized by a decrease in memory function and hippocampal alterations. Its early stage shows synaptic alterations and an increase in synaptic E/I balance and neuronal hyperactivity, resulting in induced neuron loss and reduction in hippocampal volume at late stages [51,52]. Amyloid β peptide 1–40 or 1–42 (Aβ_1–40 or 1–42_) is known as a major causative agent [53,54,55,56]. A biomarker study showed that Aβ_1–42_ accumulation signals the symptom onset of synaptic dysfunction, tau-mediated neuronal injury, brain structure, cognition and clinical function [57]. Soluble Aβ_1–40_ oligomers impair long-term potentiation and increased neuronal hyperactivity by glutamatergic/GABAergic imbalance in the hippocampus [51,52]. Long-term exposure to Aβ_1–42_ (1–3 d) impaired AMPA receptor trafficking by reducing the synaptic distribution of Ca^2+^ and calmodulin-dependent protein kinase II in cultured pyramidal neurons [58]. In contrast, the effect of soluble oligomeric assemblies of Aβ_1–42_ oligomer is more rapid, decreasing surface levels of AMPA receptors within 30 min [59].

While less is known about its toxic effects at inhibitory synapses, Aβ_1–42_ specifically binds to nicotinic α_7_ receptors [60], impairing learning-induced plasticity at GABA_A_ receptor-mediated inhibitory synapses [10,61]. Bath application of Aβ_1–42_ weakens GABA_A_ receptor-mediated synaptic currents within 10 min through GABA_A_ receptor downregulation via receptor endocytosis in slice [62], while directly blocking nicotinic α7 receptor-mediated cholinergic response within 3 min [63]. This result indicates that the disinhibited GABA_A_ receptor-mediated synaptic inhibition by Aβ leads to the hyperexcitability characteristic of AD, and might be partly related to the loss of functional GABA_A_ receptors in the AD brain [62,64]. Understanding the dynamic changes occurring during learning-promoted plasticity is necessary to identify a failure point in cognitive disorders.

#### GABA_A_ Receptor as Therapeutic Target in AD

Since Aβ weakened GABA_A_ receptor-mediated synaptic inhibition, GABA_A_ receptor agonists may improve either symptoms or progression of AD. A human AD patient showed several alterations in GABA_A_ receptor subunits including α_1_, α_2_, α_5_, β_2_, β_3_ and γ_2_ [65,66]. In cultured rat cortical neurons pre-treatment with muscimol, a GABA_A_ receptor agonist, ≥24 h prior to Aβ_1–42_ treatment inhibited Aβ_1–42_-induced neuronal apoptosis and glutamate release [67]. Moreover, chronic administration of propofol to aged (18-months old) mice also decreased Aβ_1–40_ and Aβ_1–42_ levels [68]. However, baclofen, a GABA_A_ receptor and GABA_B_ receptor agonist, failed to inhibit Aβ_1–42_ induced neuronal death [67]. Thus, selective GABA_A_ receptor activation prevents Aβ’s adverse effects on neurons.

Moreover, AD patient hippocampus showed decreased GABA_A_Rβ3 expression [64,65]. Phosphorylation in β_3_ subunit Ser^408–409^ facilitated synaptic trafficking of GABA_A_ receptor and prevented the receptor internalization, resulting in an increase in GABA_A_ receptor-mediated postsynaptic currents [29]. Recently, we reported that contextual learning rapidly strengthened GABA_A_ receptor-mediated postsynaptic currents and Ser^408–409^ phosphorylation in the β_3_ subunit, suggesting that phosphorylation underlies rapid inhibitory synaptic plasticity and contextual memory encoding [23]. While Aβ_1–42_ treatment decreased GABA_A_ receptor-mediated postsynaptic currents via receptor internalization, inhibiting GABA_A_ receptor endocytosis prevented its adverse effects [62]. Thus, controlling GABA_A_ receptor trafficking may provide a new therapeutic target in AD.

A benzodiazepine (BZD) binding site is located in the extracellular domain of the GABA_A_ receptor, at the α+/γ− interface, which modulates the GABA-induced Ch^-^ ion current [69]. AD patients show a reduction in the abundance of BZD sites in the hippocampus [70]. Baicalein (a positive allosteric modulator of the BZD site) significantly reduced Aβ production, improved cognitive function and decreased pathological features in an eight-week-old AD mouse model [68]. Moreover, our preliminary data shows that Aβ_1–42_ oligomers significantly impair the single channel current but not the number of channels in postsynaptic GABA_A_ receptors by using non-stationary fluctuation analysis, suggesting that Aβ_1–42_ oligomers act as a negative allosteric modulator [71]. Flumazenil, a silent or neutral allosteric modulator, was shown to prevent positive/negative allosteric modulator for the occupation of a binding site [72]. The hippocampus of AD patients showed a decrease in flumazenil binding, being positively correlated with hippocampal volume and memory function [73]. Thus, silent or neutral allosteric modulators may prevent adverse Aβ_1–42_ oligomer effects, improving hippocampal function at early stages of AD.

### 5.2. ASD

ASDs are a group of complex neurodevelopmental disorders characterized by repetitive behaviors and deficit of social cognitive and synaptic E/I imbalance [74]. They result from a complex interaction between genetics and the environment, with heritability estimates ranging from 40 to 80% [75,76]. Genetic studies have reported a few hundred genes linked to ASD, some encoding GABA_A_ receptor subunits, namely *GABRB3*, *GABRA5* and *GABRG3*, encoding for β_3_, α_5_ and γ_3_ subunits, respectively [74,76,77]. In particular, *GABRB3* (rs2081648 and rs1426217) presented a single-nucleotide polymorphism associated with ASD regardless of age or sex [74,78]. A deficiency of GABA_A_Rβ3 (Gabrb3) in mice reduces GABA_A_ receptor expression and enhances seizure susceptibility and autistic-like cognitive and motor deficits [30,79,80]. Indeed, ASD patients showed decreased expression of GABA_A_Rβ3s in the parietal cortex and the cerebellum [81].

While the hippocampus of ASD patients has a larger volume than that from healthy persons from childhood to adolescence [82], few studies have examined hippocampal dysfunction in ASD. Recently, an ASD patient showed a deficit in hippocampal-dependent memory, including cognitive maps or episodic memory [83,84]. In addition, the hippocampal CA2 region plays an essential role for social recognition memory [85]. Recently, there had been increasing interest is hippocampal dysfunction, synaptic alternation and relating cognition in ASD.

#### GABA_A_ Receptor as Therapeutical Target in ASD

Since genetic animal models for ASD have often shown a reduction in inhibitory neurotransmission, GABA agonists have been used as therapy [86]. *PX-RICS*^−/−^ mice (loss-of-*PX-RICS* function) exhibit ASD-like behaviors, and have reduced GABA_A_ receptor surface expression and lower mIPSC amplitude but not frequency [87]. A GABA_A_ receptor agonist (clonazepam, a positive allosteric modulator of the BZD site) improved some of its ASD-like behavioral phenotypes [87]. Other ASD mouse models (BTBR mice: Idiopathic autism; *Scn1a*^+/−^ mice: A monogenic model of ASDs [88]) also showed a reduced GABA_A_ receptor -mediated inhibition; treatment with positive allosteric modulators, either BZD or clonazepam, led to improved social and cognitive deficits [88,89]. Interestingly, a selective positive allosteric modulator of GABA_A_ receptor α_2_ and/or α_3_ subunits, L-838,417, also improved behavioral deficits in both BTBR and *Scn1a*^+/−^ mice [89]. Accordingly, clinical trials using α_2_/α_3_ selective positive allosteric modulators of GABA_A_ receptors have been developed by AstraZeneca and the National Institutes of Health [86].

In addition, a recent study reported an alteration of synaptic trafficking via phosphorylation in ASD [50,90]. The sodium valproate-induced rat ASD model shows impaired spatial memory, limited exploration, increased anxiety and reduced sociability [90], and reduced GABA_A_Rβ3 expression at different postnatal developmental stages, as well as downregulation of the phosphorylated form of the receptor subunit. This reduction facilitates receptor internalization, resulting in blocked inhibitory plasticity [50]. Thus, GABA_A_Rβ3 phosphorylation may prevent a decrease in GABA_A_ receptor expression and allow recovery from synaptic alterations and cognitive dysfunction in ASD.

### 5.3. SE

Epilepsy is group of neurological disorders characterized by a striking synaptic E/I imbalance [87]. SE is defined as seizure lasting >30 min or occurrence of ≥2 seizures without recovery of consciousness [91]. Inactivation of GABA_A_ receptors with bicuculline or picrotoxin results in epileptic seizures [92]. In addition, mutant animals lacking the β_3_ (*Gabrb3*) subunit showed neuronal hyperactivity and seizures, which led to pathologies such as AD, ASD and Angelman syndrome [30,74,93,94,95].

Interestingly, epileptic patients consistently show cognitive deficits, but their underlying basis is yet to be determined [96]. In animal studies, kainic acid-induced SE impairs hippocampal-dependent short- and long-term spatial teaching [97], suggesting there are adverse effects of SE on hippocampal cognitive function. In the hippocampus, SE induced by pilocarpine, a non-selective mAChR agonist, decreased PKC-mediated phosphorylation of β_3_ subunit Ser^408–409^ and increased binding to AP2 and GABA_A_ receptor endocytosis via dephosphorylation [98]. An acute stressor, such as foot shock or restrain, increased the performance in hippocampal-dependent tasks [99,100] and hippocampal BDNF concentration [100], while decreasing seizure susceptibility [101]. Recently, we found rapidly strengthened GABA_A_ receptor mediated synapses and phosphorylation in β_3_ subunit Ser^408–409^ immediately after a foot shock in an IA task training. Thus, we suggested that the rapid inhibitory plasticity produced by the exposure to a stressful episode might contribute to reducing seizure vulnerability and maintaining cognitive function in the hippocampus.

#### GABA_A_ Receptor as Therapeutical Target in SE

The GABA_A_ receptor is a major target of antiseizure drugs [102,103,104]. In particular, BZDs, positive allosteric modulators, are effective in improving and blocking seizures [102,103]. Temporal lobe epilepsy has been linked to a significant loss of BZD binding sites [102], and the activation of GABA_A_ receptors by various allosteric ligands is crucial for the prevention of seizures [105]. Indeed, current SE treatment guidelines recommend a stepwise anti-seizure medication treatment with up to two BZD doses within the first 5–10 min of SE onset, followed by non-BZD ASM after 10 min [106,107].

In addition, new drugs have focused on GABA_A_Rβ3 phosphorylation. Loreclezole, a subtype-selective positive allosteric modulator, increased the seizure threshold caused by a strongly potentiated recombinant GABA_A_ receptor containing a β_2_ or β_3_ subunit but not β_1_-containing receptors. Moreover, phosphorylating β_3_ subunit Ser^408–409^ by PDBu (a PKC activator) increases GABA_A_ receptor cell surface expression levels and recovers synaptic inhibition in SE [98]. Thus, rapid GABA_A_ mediated inhibitory plasticity via phosphorylation of β_3_ subunit Ser^408–409^ may prevent seizure vulnerability and improve memory function in SE patients.

### 5.4. PTSD

PTSD is an anxiety disorder that occurs following exposure to severe trauma. The lifetime prevalence of PTSD is about 10–12% in women and 5–6% in men [108]. In human and animal studies, early traumatic experience, such as maternal separation, postnatal neglect and abuse, significantly increase abnormal behavioral reaction, alternation of brain morphology and synaptic plasticity in adulthood [109,110]. PTSD is characterized by deficits in GABAergic transmission and cognitive function in the brain, in particular the hippocampus [111]. Juvenile traumatic stress induced chronic anxiety, hippocampal-dependent memory loss and alternation in some subunit expression of GABA_A_ receptor in the hippocampus [110]. In addition, several studies reported that hippocampal GABAergic dysfunction attenuated juvenile stress induced an increase in risk factor of PTSD and cognitive and synaptic plasticity impairments [112,113,114,115,116]. Recently, Torrisi et al. [117] found an impaired hippocampal synaptic plasticity specifically at CA3–CA1 synapses of trauma susceptible mice showing long-lasting PTSD-like phenotypes.

#### GABA_A_ Receptor as Therapeutical Target in PTSD

During the juvenile period, exposure of traumatic stress induced alternation in some α subunit (α_1_, α_2_, and α_5_) expression of GABA_A_ receptor in the hippocampus [110,118]. The α subunit of the GABA_A_ receptor is associated with various pharmacological properties of BZD [119]. PET (positron emission tomography) scan showed significantly reduced flumazenil binding through the cortex, hippocampus and thalamus in PTSD patients [120]. Treatment of BZD strengthened inhibitory neurotransmission by binding to the BZD site of the GABA_A_ receptor, resulting in improving anxiety and sleep disturbances in PTSD [110]. Recently, another study showed an increase in GABA_A_ receptor α_1_ subunit expression in CA1 after juvenile traumatic stress [118]. They also reported that enriched environment exposure during juvenility prevented stress-associated increase of the α_1_ subunit [118]. Thus, dysfunction of the GABA_A_ receptor α_1_ subunit may improve some PTSD symptoms. On the other hand, while the relation of the GABA_A_Rβ3 subunit gene (GABRB3) to PTSD patients has been known [121], few studies have examined a therapeutic efficacy of the β_3_ subunit. Thus, understanding of GABA_A_Rβ3 subunit in relation to PTSD will lead to the development of novel therapeutic agents.

## 6. Conclusions

Contextual learning not only induces synaptic delivery of AMPA receptors but also strengthens GABA_A_ receptor-mediated inhibitory synapses onto CA1 neurons. Several pathologies, such as AD, ASD and SE, are characterized by neuronal hyperactivity, downregulation of inhibitory neurotransmission and alterations in GABA_A_Rβ3 trafficking and phosphorylation [50,64,65,66]. Indeed, GABA_A_ receptor agonist or positive allosteric modulator can help improve some symptoms of these pathologies. However, other GABA_A_ receptor subunits (e.g., α_1_, α_2_, α_5_, β_2_, β_3_, and γ_2_) are also consistently altered in these pathologies. While the complexity of these alterations is not compatible with a simple compensatory mechanism [65,66], we believe that understanding GABA_A_ receptor trafficking would provide new therapeutic targets for these pathologies.
